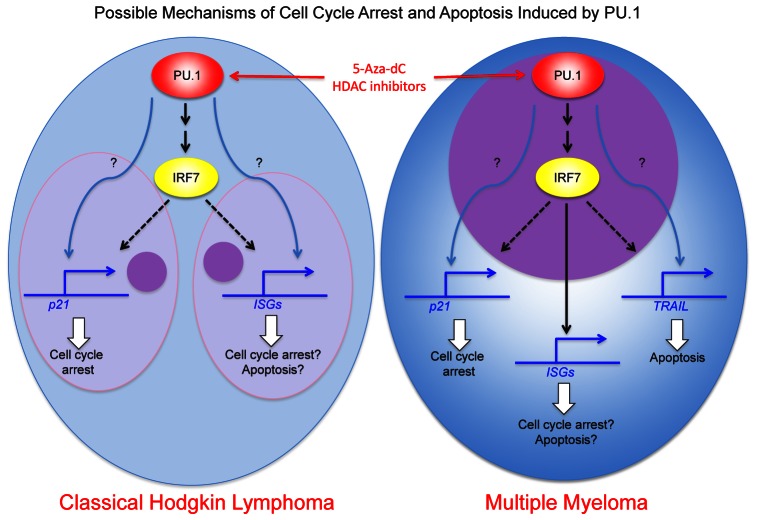# PU.1 is a tumor suppressor for B cell malignancies

**DOI:** 10.18632/oncotarget.800

**Published:** 2012-12-31

**Authors:** Yutaka Okuno, Hiromichi Yuki

**Affiliations:** Department of Hematology, Kumamoto University of Medicine, Kumamoto, Japan; Department of Hematology, Kumamoto University of Medicine, Kumamoto, Japan

PU.1 is a critical transcription factor for differentiation of both myeloid and lymphoid cells. *PU.1* knockout mice are embryonic lethal or die soon after birth, and those mice do not have granulocytes, monocytes/macrophages, or B cells [1, 2]. PU.1 is expressed in granulocytes, monocytes/macrophages, and B cells, but not in erythrocytes, megakaryocytes, or T cells. It was recently shown that PU.1 is an essential transcription factor for T_H_9 cell differentiation using a conditional mouse knockout model [3]. Collectively, these data show that PU.1 is essential for the differentiation of most hematopoietic lineages. In contrast, PU.1 expression in conditional knockout mice of a 14 kb upstream enhancer element (URE) of PU.1 gene reduced to 20% of wild type, and those mice developed acute myeloid leukemia (AML) and B-CLL-like disease [4]. These data show that PU.1 expression is tightly regulated in specific lineage cells and the decreased PU.1 expression results in various hematological malignancies.

Meanwhile, PU.1 function is not well understood in B cells. In the early lymphoid commitment stage, the loss of PU.1 expression leads to complete failure of lymphoid differentiation in both B and T cells. In late B cell development, the loss of PU.1 expression induced by a CD19-Cre system had no effect on B cell differentiation [5], suggesting that PU.1 may not be necessary for mature B cell differentiation. However, this does not explain the facts that in conventional PU.1 knockout mice B cells are defective, but T cells are not, and that decreased PU.1 expression (noted above) induced B-CLL-like disease. These observations prompted us to try to elucidate the function of PU.1 in B cell malignancies, starting with multiple myeloma, a malignancy of plasma cells. Most myeloma cell lines have lost PU.1 expression, while primary myeloma cells from patients have decreased PU.1 expression and normal plasma cells have relatively high levels. We have demonstrated that downregulation of PU.1 in myeloma cell lines is caused by an epigenetic mechanism. In addition, conditional expression of PU.1 using a Tet-off system induced growth arrest and apoptosis in myeloma cell lines [6]. This suggests that PU.1 may be a tumor suppressor for multiple myeloma. In another B cell lymphoid malignancy, classical Hodgkin lymphoma, PU.1 is also downregulated through promoter methylation. Therefore, we speculated that PU.1 might also be a tumor suppressor for classical Hodgkin lymphoma. Thus, we introduced conditional PU.1 expression in the classical Hodgkin lymphoma cell lines, L428 and KM-H2 using the same Tet-off system. Conditional PU.1 expression induced complete growth arrest and apoptosis in these cell lines [7]. We also transplanted the cell lines in immunodeficient mice, and observed that tetracycline withdrawal induced growth arrest (or shrinking of tumors) and prolonged survival. We also demonstrated that a lentiviral system containing PU.1 could induce apoptosis in primary Hodgkin lymphoma purified from patients. Together, these data suggest that PU.1 is a tumor suppressor in classical Hodgkin lymphoma cells. In addition, we treated six classical Hodgkin lymphoma cell lines with 5'-aza-2'-deoxycytidine and/or an HDAC inhibitor, trichostatin A. These treatments induced PU.1 expression, growth arrest, and apoptosis in all cell lines tested. These data suggest that upregulation of PU.1 by demethylation agents and/or HDAC inhibitors may be a promising therapy for classical Hodgkin lymphoma.

We are now examining the mechanisms for growth arrest and apoptosis induced by PU.1 in classical Hodgkin lymphoma cells and myeloma cell lines. We showed previously that TRAIL is upregulated in myeloma cell lines through direct transactivation by PU.1 and plays a role in apoptosis in those cells [8]. In contrast, TRAIL is initially expressed at high levels in Hodgkin lymphoma and PU.1 does not induce upregulation of TRAIL. In L428 cells, p21 is upregulated by PU.1 induction, and knockdown of p21 rescues growth arrest induced by PU.1, suggesting p21 is involved in PU.1 induced growth arrest. This is also observed with the myeloma cell line, U266. However, p21 is not upregulated following PU.1 induction in another Hodgkin lymphoma cell line, KM-H2, or a myeloma cell line, KMS12PE. Therefore, growth arrest mechanisms do not appear to be the same among all Hodgkin lymphoma and myeloma cell lines. We performed DNA microarray analysis to examine highly upregulated and downregulated genes following PU.1 induction in Hodgkin and myeloma cell lines. Among those genes examined, IRF7, which is important for interferon cascade, is highly upregulated in all four cell lines. Because IRF1 is known to directly transactivate *p21*, IRF7 may also up-regulate p21. Our data show that many interferon stimulated genes (ISGs) are highly upregulated after PU.1 induction in L428 and U266 cells in parallel with p21 upregulation, but not in KM-H2 and KMS12PE cells, which do not upregulate p21. Therefore, in these cells, other tumor suppressors, including p15, p16, and p73, but not p21 may be transactivated by PU.1. Identifying PU.1 downstream targets that induce growth arrest and apoptosis may serve as a guide to new targeted therapies for Hodgkin lymphoma and multiple myeloma.

**Figure:**